# Supplementary material for: When South Meets North: A Joint Contact Zone Coinciding With Environmental Gradients in Three Boreal Tree Species
Source: Mol Ecol. 2026 Apr 12;35(8):e70344. doi: 10.1111/mec.70344 (PMC13071491; doi:10.1111/mec.70344)
Supplement: Supplementary file 2 — Table S1: Summary of candidate SNPs and genes identified by genome scans and GEA analyses across all species. For total number of SNPs and genes, proportion compared to full data is given in brackets. Table S2: Coefficients of overlap for candidate genes from the different genome scans and GEA analyses, calculated as the intersect across datasets divided by the minimum dataset size. P: pcadapt, X: XTX, G: GEA. Table S3: Coefficients of overlap of GO terms for candidate genes and background genes (from SNPs in initial VCF file) in all species and pairwise comparisons between them (df = 1, p‐values < 1.3e‐51). Table S4: Proportion of clinal alleles in different allele sets (neutral and selection outliers) and the adjusted p‐values (Bonferroni) from hypergeometric test for enrichment compared to all dataset are given inside brackets. Significantly enriched datasets are depicted in bold. [file MEC-35-e70344-s002.docx]

**SUPPLEMENTARY TABLES**

**Table S1:**  Summary of candidate SNPs and genes identified by genome scans and GEA analyses across all species. For total number of SNPs and genes, proportion compared to full data is given in brackets.

| **Analyses** | ***P. abies*** | | ***B. pendula*** | | ***P. sylvestris*** | |
| --- | --- | --- | --- | --- | --- | --- |
|  | **SNPs** | **genes** | **SNPs** | **genes** | **SNPs** | **genes** |
| *pcadapt* | 39 | 17 | 119 | 46 | 42 | 21 |
| X^T^X | 806 | 363 | 137 | 51 | 92 | 43 |
| Annual Mean Temperature (Bio1) | 68 | 32 | 121 | 48 | 50 | 29 |
| Mean Diurnal Range (Bio2) | 14 | 10 | 29 | 16 | 33 | 21 |
| Isothermality (Bio3) | 35 | 27 | 96 | 42 | 43 | 23 |
| Temperature Seasonality (Bio4) | 62 | 32 | 86 | 43 | 29 | 18 |
| Max Temperature of Warmest Month (Bio5) | 24 | 18 | 71 | 33 | 37 | 25 |
| Min Temperature of Coldest Month (Bio6) | 75 | 36 | 99 | 45 | 39 | 22 |
| Temperature Annual Range (Bio7) | 48 | 24 | 32 | 22 | 25 | 14 |
| Mean Temperature of Wettest Quarter (Bio8) | 12 | 10 | 0 | 0 | 13 | 8 |
| Mean Temperature of Driest Quarter (Bio9) | 26 | 17 | 8 | 8 | 25 | 8 |
| Mean Temperature of Warmest Quarter (Bio10) | 27 | 19 | 80 | 37 | 35 | 24 |
| Mean Temperature of Coldest Quarter (Bio11) | 80 | 36 | 113 | 46 | 39 | 22 |
| Annual Precipitation (Bio12) | 13 | 9 | 2 | 2 | 38 | 21 |
| Precipitation of Wettest Month (Bio13) | 16 | 12 | 2 | 2 | 2 | 1 |
| Precipitation of Driest Month (Bio14) | 11 | 8 | 2 | 2 | 25 | 13 |
| Precipitation Seasonality (Bio15) | 13 | 10 | 0 | 0 | 46 | 28 |
| Precipitation of Wettest Quarter (Bio16) | 14 | 11 | 3 | 3 | 13 | 5 |
| Precipitation of Driest Quarter (Bio17) | 10 | 6 | 2 | 2 | 31 | 11 |
| Precipitation of Warmest Quarter (Bio18) | 18 | 13 | 1 | 1 | 10 | 3 |
| Precipitation of Coldest Quarter (Bio19) | 5 | 4 | 2 | 2 | 36 | 18 |
| Unique Total | 999 (0.17%) | 495 (1.77%) | 163 (0.31%) | 66 (3.05%) | 197 (0.22%) | 96 (1.65%) |
| Unique Total GEA (all Bio) | 300 (0.05%) | 183 (0.65%) | 142 (0.27%) | 61 (2.82%) | 142 (0.16%) | 76 (1.30%) |

**Table S2:** Coefficients of overlap for candidate genes from the different genome scans and GEA analyses, calculated as the intersect across datasets divided by the minimum dataset size. P: *pcadapt*, X: X^T^X, G: GEA.

| **Overlap Coefficient** | ***P. abies*** | ***B. pendula*** | ***P. sylvestris*** |
| --- | --- | --- | --- |
| **P∩X∩G** | 0.76 | 0.93 | 0.57 |
| **P∩X** | 1.00 | 0.98 | 0.86 |
| **X∩G** | 0.28 | 0.90 | 0.56 |
| **P∩G** | 0.76 | 0.96 | 0.67 |

**Table S3:** Coefficients of overlap of GO terms for candidate genes and background genes (from SNPs in initial VCF file) in all species and pairwise comparisons between them (df =1, *P*-values < 1.3e-51).

| **Comparison** | **GO terms** | **Common** | **Total** | **Ratio (95% CI)** | **χ^2^** |
| --- | --- | --- | --- | --- | --- |
| All species | candidate | 83 | 1496 | 0.06 (0.04,0.07) | 1181 |
|  | all | 2773 | 7919 | 0.35 (0.34,0.36) | 710 |
| Pabies_Bpendula | candidate | 166 | 1305 | 0.13 (0.11,0.15) | 724 |
|  | all | 3219 | 7638 | 0.42 (0.41,0.43) | 188 |
| Bpendula_Psylvestris | candidate | 101 | 635 | 0.16 (0.13,0.19) | 294 |
|  | all | 2810 | 5936 | 0.47 (0.46,0.49) | 17 |
| Pabies_Psylvestris | candidate | 187 | 1340 | 0.14 (0.12,0.16) | 695 |
|  | all | 5071 | 7818 | 0.65 (0.64,0.66) | 690 |

**Table S4:** Proportion of clinal alleles in different allele sets (neutral and selection outliers) and the adjusted *P-*values (Bonferroni) from hypergeometric test for enrichment compared to all dataset are given inside brackets. Significantly enriched datasets are depicted in bold.

| **Allele Set** | ***P. abies*** | ***B. pendula*** | ***P. sylvestris*** |
| --- | --- | --- | --- |
| Neutral | 0.0105 (0.099) | 0.0096 (1.000) | **0.0134 (1.36e-11)** |
| *pcadapt* | **1.000 (4.80e-21)** | **1.000 (1.06e-177)** | **0.692 (2.54e-23)** |
| X^T^X | **0.932 (0.00e+0)** | **1.000 (1.45e-206)** | **0.724 (1.37e-51)** |
| Annual Mean Temperature (Bio1) | **0.985 (2.51e-76)** | **1.000 (7.00e-181)** | **0.940 (4.89e-64)** |
| Mean Diurnal Range (Bio2) | 0.214 (3.50e-01) | **1.000 (6.38e-27)** | **0.879 (3.95e-39)** |
| Isothermality (Bio3) | **0.743 (4.06e-25)** | **0.989 (4.10e-138)** | **0.930 (2.60e-54)** |
| Temperature Seasonality (Bio4) | **0.903 (8.43e-50)** | **1.000 (3.25e-126)** | **1.000 (1.28e-35)** |
| Max Temperature of Warmest Month (Bio5) | **0.500 (1.55e-11)** | **0.986 (1.20e-95)** | **0.919 (7.48e-46)** |
| Min Temperature of Coldest Month (Bio6) | **0.933 (5.80e-66)** | **1.000 (2.74e-146)** | **1.000 (1.02e-51)** |
| Temperature Annual Range (Bio7) | **0.854 (7.66e-46)** | **1.000 (6.79e-30)** | **0.960 (6.04e-28)** |
| Mean Temperature of Wettest Quarter (Bio8) | 0.000 (1.000) | 0.000 (1.000) | **0.769 (5.66e-10)** |
| Mean Temperature of Driest Quarter (Bio9) | **0.269 (8.40e-05)** | **1.000 (3.55e-06)** | **0.840 (5.08e-26)** |
| Mean Temperature of Warmest Quarter (Bio10) | **0.407 (1.72e-08)** | **0.975 (1.11e-105)** | **0.914 (1.97e-42)** |
| Mean Temperature of Coldest Quarter (Bio11) | **0.975 (1.17e-87)** | **1.000 (3.30e-168)** | **1.000 (1.03e-51)** |
| Annual Precipitation (Bio12) | 0.000 (1.000) | 0.000 (1.000) | **0.815 (3.00e-36)** |
| Precipitation of Wettest Month (Bio13) | 0.000 (1.000) | 0.000 (1.000) | 0.00 (1.00) |
| Precipitation of Driest Month (Bio14) | 0.000 (1.000) | 0.000 (1.000) | **0.760 (1.20e-18)** |
| Precipitation Seasonality (Bio15) | 0.154 (1.000) | 0.000 (1.000) | **0.934 (2.12e-57)** |
| Precipitation of Wettest Quarter (Bio16) | 0.000 (1.000) | 0.000 (1.000) | **0.466 (8.54e-03)** |
| Precipitation of Driest Quarter (Bio17) | 0.000 (1.000) | 0.000 (1.000) | **0.806 (2.05e-24)** |
| Precipitation of Warmest Quarter (Bio18) | 0.000 (1.000) | 0.000 (1.000) | **0.600 (7.24e-03)** |
| Precipitation of Coldest Quarter (Bio19) | 0.000 (1.000) | 0.000 (1.000) | **0.805 (3.06e-26)** |
