# Supplementary material for: When South Meets North: A Joint Contact Zone Coinciding With Environmental Gradients in Three Boreal Tree Species
Source: Mol Ecol. 2026 Apr 12;35(8):e70344. doi: 10.1111/mec.70344 (PMC13071491; doi:10.1111/mec.70344)
Supplement: Supplementary file 3 — Data S1: mec70344‐sup‐0003‐Supinfo.pdf. [file MEC-35-e70344-s003.pdf]

When South meets North: a joint contact zone coinciding with environmental gradients in three boreal tree species

Pilar Herrera-Egoavil[1], J. Luis Leal[1,2], Qiujie Zhou[1,3], Pascal Milesi[1,3], Martin Lascoux[1], Burçin Yildirim[1,4,*]

1. Department of Ecology and Genetics, EBC, Uppsala University, Sweden
2. Department of Zoology, Stockholm University, Sweden
3. SciLifeLab Uppsala, Sweden
4. Department of Cell and Molecular Biology, Uppsala University, Sweden

*Author for correspondence: burcinyildirim92@gmail.com, burcin.yildirim@icm.uu.se

Supplementary Methods

1. Dataset and SNP calling specifications

Picea abies (Norway spruce)

The trees for *Picea abies* were genotyped using an exome-capture target resequencing strategy (J. Chen et al., 2019; Z.-Q. Chen et al., 2021; 40,018 diploid probes with 120bp length Vidalis et al., 2018). From the initial dataset of 1758 trees with confirmed Swedish origin (Li et al., 2022), we chose 154 trees to get a sampling similar to two other species (see below), between latitudes 56°N and 67°N (Table 1).

We checked the quality of the raw reads with FastQC (v.0.11.9; Andrews, 2010) and trimmed them with Trimmomatic (Bolger et al., 2014) before mapping with BWA-MEM (v.0.7.17, Li, 2013) against the reference genome for *Picea abies* (Pabies1.0; Nystedt et al., 2013). Alignment files were then filtered and optical PCR duplicates were removed using the PICARD suites (v.2.27.5; Broad Institute, 2019). Genotype identification and SNP calling were carried out with GATK tools HaplotypeCaller (for each sample) and GenotypeGVCFs (jointly across all samples). The raw VCF file later filtered for biallelic SNPs and hard-filtered to remove low-quality SNPs with the following criteria: i) Variant confidence (QD) < 2.0, ii) Root Mean Square of the mapping quality (MQ) < 40.0, iii) Odds Ratio for strand bias (SOR) > 3.0, iv) Rank sum test for mapping qualities (MQRankSum) < -12.5, v) Rank Sum Test for site position within reads (ReadPosRankSum) < -8.0, QUAL < 20.0, and vi) Phred-scaled probability of the strand bias (FS) > 60.0. SNPs with sites with > 50% missingness were filtered out of the dataset. We also used the rCNV R package to remove pseudo-SNPs stemming from multicopy regions (Karunarathne et al., 2023). By using Z-score and chi-square tests with the assumption of p=0.5 for allele occurrence probability, we identified 120,236 SNPs lying in putative paralogous and CNV regions, which were subsequently filtered out. The SNPs in final dataset were functionally annotated using the reference genome for *P. abies* (Pabies1.0; Nystedt et al., 2013) and SNPEff (v.4.3t; Cingolani et al., 2012) and were classified as “intergenic”, ”intronic”, ”nonsynonymous” and ”synonymous”. The number of SNPs in each dataset and functional categories are given in Table 2.

Table 1: Population data for *P. abies*

**Longitude**	**Latitude**	**Sample size**	**Population Name**
22.26	66.59	13	PA12
21.30	65.77	13	PA11
20.92	64.68	13	PA10
19.95	64.18	13	PA09
17.67	63.48	13	PA08
17.01	62.43	13	PA07

16.51	61.49	13	PA06
17.67	60.04	12	PA05
16.89	59.519	12	PA04
15.13	58.34	13	PA03
14.66	56.98	13	PA02
13.87	56.61	13	PA01

Table 2: Number of SNPs for each species in different datasets and functional categories. The putatively neutral dataset includes SNPs in intergenic, intronic and synonymous regions.

	P. abies	***B. pendula***	***P. sylvestris***
Final full	597,182	51,768	86,722
Intergenic	112,161 (18.80%)	0	26,315 (30.3%)
Intron	220,697 (36.9%)	14,135 (27.03%)	34,100 (39.32%)
Synonymous	82,360 (13.8%)	9,549 (18.45%)	5,781 (6.67%)
Nonsynonymous	124,462 (20.8%)	9,396 (18.15%)	10,206 (11.77%)
Neutral	415,216 (69.53%)	23,684 (45.75%)	66,196 (76.33%)

Betula pendula (Silver birch)

For *Betula pendula*, leaves were collected from 150 trees from 12 populations distributed along a latitudinal gradient in Sweden (from 56°N to 68°N; Table 3), and DNA was isolated according to the protocol of Leal et al. (2024). Trees were genotyped using exome capture sequencing (Illumina pair-end short-reads, 150 bp) performed at IGA Technology Services (Udine, Italy) using a 10K probe set defined by Milesi et al. (2024).

Quality checking of raw reads and trimming was performed with FastQC (v.0.11.9; Andrews, 2010) and Trimmomatic (Bolger et al., 2014) before mapping with BWA-MEM (v.0.7.17, Li, 2013) against the reference genome for *B. pendula* (Bpev01; Salojärvi et al., 2017). Using PICARD suites (v.2.27.5; Broad Institute, 2019), alignment files were filtered and optical PCR duplicates were removed. We performed joint variant calling using GATK's GVCF workflow to obtain raw VCF files. The variants were filtered to biallelic SNPs and subsequently hard-filtered, with the following criteria: i) QD < 4.0, ii) MQ < 55.0, iii) SOR > 3.0, MQRankSum < -10.0, MQRankSum < -5.0 and > 5.0, FS > 20.0. Additionally, the variants were filtered for genotype read depth DP < 4.0 to avoid bias when calculating variant DP for some haplotypes and excluded sites with more than 50% missingness. We also flagged and filtered 5,128 pseudo-SNPs considered as coming from misalignment of putative paralogous or multi-copy regions using the rCNV R package, with similar parameters as for *P. abies*. The annotated file was generated using the reference genome for *B. pendula* and SNPEff (Table 2).

Table 3: Population data for *B. pendula*

**Longitude**	**Latitude**	**Sample size**	**Population Name**
21.79	68.02	7	BP12
21.22	65.5	12	BP11
21.07	64.53	12	BP10
19.97	63.91	9	BP09
18.4	63.13	4	BP08
17.35	62.16	7	BP07
16.65	61.03	7	BP06
16.67	60.1	18	BP05
17	59.48	23	BP04
15.1	58.41	20	BP03
14.55	56.75	13	BP02
13.88	56.02	18	BP01

Pinus sylvestris (Scots pine)

The Scots pine (Pinus sylvestris) data have been sampled and sequenced (genotyping-by-sequencing) by Bruxaux et al., 2024. The raw data for 351 individuals from 10 Swedish populations (Table 4) were downloaded from NCBI database (accession number PRJNA976641) using *sratools* (https://github.com/ncbi/sra-tools). We assessed the quality of the raw reads with FastQC (Andrews, 2010) and trimmed the first five bases corresponding to the enzyme recognition site using *seqtk* (https://github.com/lh3/seqtk). Clean reads were mapped to *P. tabuliformis* reference genome (V1.0; Niu et al., 2022) using the BWA-MEM algorithm with default parameters (Li & Durbin, 2009). BAM files were obtained with samtools (Li et al., 2009). Mate information between each read was fixed, and BAM files were cleaned (soft clipping and setting mapping quality for unmapped reads) via Picard (https://broadinstitute.github.io/picard/). Variants were identified, and individual genotype VCF (GVCF) files were combined and jointly genotyped using the *HaplotypeCaller*, *GenomicsDBImport*, and *GenotypeGVCFs* tools of GATK, respectively (McKenna et al., 2010). From the resulting VCF files, we only extracted biallelic SNPs. We applied hard filtering according to following criteria: i) MQ < 55 ii) FS > 20 and SOR > 3 iii) Variant confidence (QD) < 4 iv) MQRankSum < -4 and > 4, v) ReadPosRankSum < -2 and > 2, vi) overall read depth from all samples DP < 4. Additionally, we filtered out sites with more than 50% missing data. Finally, we filtered out 9406 variants, potentially overlapping with copy number variations (CNVs) using the rCNV R package. These SNPs were found by the excess of heterozygotes in combination

with the Z-score test for allele occurrence probability p = 0.5 (the significance threshold for Z-score test is modified to 0.05/sample_size). The SNPs in the final dataset were functionally annotated based on the annotation available for the reference genome using ANNOVAR (Wang et al., 2010) (Table 2).

Table 4: Population data for *P. sylvestris*

**Longitude**	**Latitude**	**Sample size**	**Population Name**
22.8	68.2	48	PS10
19.45	67.84	29	PS09
22.7	66.88	29	PS08
19.2	65.58	33	PS07
16.39	65.39	10	PS06
19.5	64.3	47	PS05
16.2	63.1	16	PS04
14.3	60.5	37	PS03
15.8	59.9	58	PS02
14	57.4	44	PS01

2. Population Structure

We used two model-based ADMIXTURE (Alexander et al., 2009), TESS3 (Caye et al., 2016) and a dimensionality reduction (Principal Component Analysis-PCA) approach to examine the population structure for each tree species. ADMIXTURE is a non-spatial model, while TESS3 is spatially explicit, which can be better to detect ancestry components when divergence in ancestral populations is low (Durand et al., 2009). For these analyses, we used putatively neutral datasets after filtering out sites in high linkage disequilibrium (r^2 > 0.2) and singletons using PLINK (Chang et al., 2015). For each species, ADMIXTURE was run with ten-fold cross-validation. We ran the R implementation of TESS3 with the assumptions of one to ten ancestral populations (K) and each K replicated 20 times. For dimension reduction, we used the PCA function of PLINK (Chang et al., 2015), which extracts principal components of the variance-standardized relationship matrix.

We defined climatic zones to assess whether the main genetic clusters of species correspond to shifts in environmental conditions across Sweden. We obtained 19 bioclimatic variables from the CHELSA database (v.1.2, http://chelsa-climate.org/, 30 arc-second resolution). The values for each bioclimatic variable corresponded to geographic coordinates across Sweden, with a 0.1-degree increment in both latitude and longitude. A principal component analysis (PCA) was performed on these data using the 'PCA' function from the R package *FactoMineR* (Lê et al.,

2008), followed by a hierarchical clustering approach using the 'HCPC' function from the same package. The optimal number of clusters was determined based on the minimum inertia gain between increasing cluster numbers.

3. Spatial Patterns of Gene Flow

To identify spatial patterns of gene flow across populations, we estimated the effective migration surfaces using the Python package FEEMS (Marcus et al., 2021). FEEMS constructs a spatial grid covering the geographic distribution of samples, and populations are assigned to the nearest node of this underlying grid. Sample coordinates and pairwise genetic distances are used to identify regions in the graph with faster or slower change in genetic similarities than predicted under a spatially homogeneous isolation-by-distance model. During the analysis, FEEMS uses a smoothing parameter, λ, to control how much emphasis is given to local structure and it can be determined with leave-one-out cross validation. We tested for 20 lambda values from 1×10^{-8} to 1×10^{2} and chose the λ value with the lowest cross-validation error to calculate the migration surfaces. This analysis is performed with the full SNP dataset after filtering out low-frequency alleles (MAF<0.01).

4. Barriers to Gene Flow

In order to detect whether there are gene flow barriers between the Northern and Southern genetic clusters of species, we used the software RIDGE (Burban et al., 2024). RIDGE employs Approximate Bayesian Computation (ABC) with random forest (RF) to classify loci as barriers between two populations. The general workflow is as follows: First, it simulates four demographic models: Strict Isolation (SI), Isolation with Migration (IM), Secondary Contact (SC), and Ancestral Migration (AM). In addition to demography, RIDGE incorporates heterogeneity in effective population size (N) along the genome due to linked selection and heterogeneity in effective migration (M) due to selection at barrier loci. These demographic models are combined with four genomic models (homo-N [1N], hetero-N [2N], homo-M [1M], hetero-M [2M]), leading to a total of 14 demographic-genomic models (no migration genomic model is considered for strict isolation). In total, these models contain 12 parameters: eight demographic (Na, N1, N2, Tsplit, Tam, Tsc, Mcur, Manc) and four genomic (two beta distribution parameters for N variation, two proportions of barriers under current and ancestral migration) (Table 5). Simulations based on these models are used to produce a reference table, training a random forest that generates weights and parameter estimates for each model according to their fit to the observed dataset. The ABC inference is performed based on both within-population summary statistics (number of SNPs, π, Watterson's θ, Tajima's *D*) and between-population statistics (F_{ST}, D*xy*, D*a*, the proportion of shared and fixed polymorphisms – *ss* and *sf*) calculated and compared for simulated and observed datasets. Second, RIDGE constructs a hypermodel where the posterior distribution of each parameter is obtained as a weighted average over the 14 models. Note that RIDGE does not select a single “correct” model, which can be arbitrary if multiple models explain the data equally well.

As RIDGE calculates summary statistics for non-overlapping windows along the genome, we chose a window size of 10 kb for *P. abies* and *B. pendula*, and 100 kb for *P. sylvestris*, based

on the SNP density in our datasets. The mutation rate for *P. abies* was fixed to 2.75×10^{-8} per site per generation (J. Chen et al., 2019), 9.5×10^{-9} per site per generation for *B. pendula* (Salojärvi et al., 2017), and 1.4×10^{-8} per site per generation for *P. sylvestris* (Pyhäjärvi et al., 2019). The recombination rate was set to 1×10^{-9} per site per generation for *P. abies*, 1×10^{-8} for *B. pendula* (Jaramillo-Correa et al., 2010), and 0.7×10^{-9} for *P. sylvestris* (Pyhäjärvi et al., 2019). RIDGE was run in its scan mode for other parameters to suggest prior values. We ran RIDGE multiple times using both suggested priors and priors from the literature, selecting the best run based on the goodness of fit between priors and posteriors (Table 6). This analysis was conducted with the full dataset.

Table 5: Parameter explanations of RIDGE

**Parameter name**	**Explanation**
Na	Ancestral effective population size
N1	Effective population size of population 1
N2	Effective population size of population 2
Tsplit	Split time of two populations (in generations)
Tam	Time ancestral migration ceases (in generations)
Tsc	Time of secondary contact (in generations)
Mcur	Current migration rate (in 4*N*mcur)
Manc	Ancestral migration rate (in 4*N*manc)
α	Shape 1 parameter of beta distribution
β	Shape 2 parameter of beta distribution
Qcur	Proportion of barriers under current migration
Qanc	Proportion of barriers under ancestral migration

Table 6: Prior values of RIDGE runs for each species

**Parameter**	***P. abies***	***B. pendula***	***P. sylvestris***
Nref (rescaling value)	4500	90000	1700
Nmin	100	5000	35
Nmax	10000	200000	3500
Tsplit min	50	0	35
Tsplit max	10000	180000	3500
Mmin	1	1	1

Mmax	500	500	1000
Pbarrier max	0.2	0.2	0.2
Mutation rate	2.75×10^{-8}	9.5×10^{-9}	1.4×10^{-8}
Recombination rate	1×10^{-9}	1×10^{-8}	0.7×10^{-9}

5. Analyses of local adaptation

5.1. Genomes Scans: PCADAPT and X^TX

We examined genomic signatures of local adaptation using the PCADAPT (Luu et al., 2017), which assumes that most SNP variation along principal component axes arises from population structure. It first performs PCA on the centered and scaled genotype matrix and second, computes test statistics and *P*-values based on the correlations between SNPs and the first K principal components (PCs). Values with extreme values are considered outliers and presumed to be SNPs under selection. We determined the number of principal components retained for population structure by controlling the percentage of variance explained by each 20 PC. For all species and given the weak population structure, K=2 was chosen as the best K. The PCA loadings were checked to avoid bias on an observed pattern caused by a small region with high linkage disequilibrium, thereby minimising false positives. We applied Bonferroni correction to the *P*-values obtained from the PCADAPT analyses for all species to account for multiple testing and identify outliers. A SNP was considered an outlier if its adjusted *P*-values > alpha (alpha < 0.01).

We also performed a population-based approach for genome scan analyses, using Bayenv2, which identifies highly differentiated SNPs based on the X^TX statistics (Coop et al., 2010; Günther & Coop, 2013). To control for a general relationship between populations, we estimated with Baypass (Gautier, 2015) a variance-covariance matrix of allele frequencies from a set of putatively neutral SNPs of 210,400, 9,420 and 38,538 for *P. abies*, *B. pendula* and *P. sylvestris.* This matrix is used as a null model when running Bayenv2, against an alternative model that assumes a linear relationship between a transformed allele frequency at a particular locus and an environmental variable of interest.

We obtained the result of X^TX statistics, which highlights loci that deviate strongly from an expected pattern of population structure. We plotted the X^TX statistics distribution and their modes (12, 12 and 10 for *P. abies*, *B. pendula* and *P. sylvestris*, respectively), which reflected the number of populations tested for each species and set a threshold. Thus, SNPs were considered significant only if their X^TX value was higher than $\overline{X^T X}$ + 4*SD for *P. abies* and *P. sylvestris* and $\overline{X^T X}$ + 3*SD for *B. pendula*.

5.2. Genotype-Environment Association (GEA) analyses: Bayes Factor

We also perform genotype-environment associations to detect significant correlations between allele frequencies and the 19 bioclimatic variables from the CHELSA dataset used before to define the climatic zones. The environmental variables were transformed, thus ensuring they are, along with the allele frequencies, in the same frame of reference. Bayes Factor (BF) is calculated to provide more support for the alternative model at a specific locus (Coop et al., 2010). Spearman's and Pearson's correlations were also obtained as alternative tests to the BF. We filtered based on BF and Spearman's *rho*, as suggested by Günther & Coop (2013), to increase the power to detect linear and non-linear relationships. Thus, we apply the following filtering for each environmental variable: a SNP was considered to be in high correlation with an environmental variable if i) its log10(BF) > 2 (decisive, according to Kass & Raftery, 1995), and ii) its absolute value of Spearman's *rho* was in the top 1% quantile.

5.3. Overlap across datasets: candidate genes, GO terms

We calculated the overlap of candidate genes detected across the genome scans and GEA analyses for each species. We calculated the overlap coefficient as the intersection between the datasets divided by the minimum dataset size for each analysis (PCADAPT, X^TX and GEA). We also compared the proportions of GO terms that overlapped between the candidate genes and the full gene dataset (background genes). We assessed whether the overlaps were significantly higher/lower than expected by chance using "prop.test" of the *stats* R package (R Core Team, 2025).

5.4 Enrichment analyses of GO terms

The list of candidate genes and associated GO terms was analysed against a larger dataset of all genes (background genes) and associated GO terms of all SNPs from the initial VCF file used in this study. Thus, 24,640, 2,089 and 5,193 background genes were used for *P. abies*, *B. pendula* and *P. sylvestris*, respectively. The GO terms were enriched if the FDR-adjusted Fisher's exact test *P*-value < 0.01. The enriched GO terms were summarised with rrvgo (Sayols, 2023), which collapses redundant terms across hierarchical levels using *Arabidopsis thaliana* as a reference for GO terms (org.At.tair.db, v.3.21.0, Carlson, 2025).

6. Used Modules/Software and versions

- sratools v.3.0.7
- FastQC v.0.11.9
- MultiQC v.1.22.2
- seqtk 1.2-r101
- bwa v.0.7.17
- samtools v.1.12
- Picard v.2.27.5
- GATK v.4.3.0.0

- PLINK v.1.9
- VCFTools v.0.1.16
- rCNV v.1.3.0
- ADMIXTURE v.1.3
- tess3r v.1.1.0
- FEEMS v.1.0.1
- SnpEff v.4.3
- FactoMineR v.2.11
- PCAdapt v. 4.4.0
- Bayenv2
- hzar v.0.2.5
- gffread v.0.12.7
- InterProScan v.5.62-94.0
- DIAMOND v.2.1.9
- topGO v.2.58.0
- rrvgo v.1.18.0
- stats v. 4.5.0

References

Alexander, D. H., Novembre, J., & Lange, K. (2009). Fast model-based estimation of ancestry in unrelated individuals. *Genome Research*, *19*(9), 1655–1664. https://doi.org/10.1101/gr.094052.109

Andrews, S. (2010). *FastQC: a quality control tool for high throughput sequence data.* [Computer software]. http://www.bioinformatics.babraham.ac.uk/projects/fastqc

Bolger, A. M., Lohse, M., & Usadel, B. (2014). Trimmomatic: A flexible trimmer for Illumina sequence data. *Bioinformatics*, *30*(15), 2114–2120. https://doi.org/10.1093/bioinformatics/btu170

Broad Institute, G. R. (2019). *Picard Toolkit* [Computer software]. https://broadinstitute.github.io/picard/

Bruxaux, J., Zhao, W., Hall, D., Curtu, A. L., Androsiuk, P., Drouzas, A. D., Gailing, O., Konrad,

H., Sullivan, A. R., Semerikov, V., & Wang, X. (2024). Scots pine – panmixia and the elusive signal of genetic adaptation. *New Phytologist*, *243*(3), 1231–1246. https://doi.org/10.1111/nph.19563

Burban, E., Tenaillon, M. I., & Glémin, S. (2024). RIDGE , a tool tailored to detect gene flow barriers across species pairs. *Molecular Ecology Resources*, *24*(4), e13944. https://doi.org/10.1111/1755-0998.13944

Carlson, M. (2025). *GO.db: A set of annotation maps describing the entire Gene Ontology* (Version 3.21.0) [Computer software]. http://bioconductor.org/packages/GO.db/

Caye, K., Deist, T. M., Martins, H., Michel, O., & François, O. (2016). TESS3: Fast inference of spatial population structure and genome scans for selection. *Molecular Ecology Resources*, *16*(2), 540–548. https://doi.org/10.1111/1755-0998.12471

Chang, C. C., Chow, C. C., Tellier, L. C., Vattikuti, S., Purcell, S. M., & Lee, J. J. (2015). Second-generation PLINK: Rising to the challenge of larger and richer datasets. *GigaScience*, *4*(1), 7. https://doi.org/10.1186/s13742-015-0047-8

Chen, J., Li, L., Milesi, P., Jansson, G., Berlin, M., Karlsson, B., Aleksic, J., Vendramin, G. G., & Lascoux, M. (2019). Genomic data provide new insights on the demographic history and the extent of recent material transfers in Norway spruce. *Evolutionary Applications*, *12*(8), 1539–1551. https://doi.org/10.1111/eva.12801

Chen, Z.-Q., Zan, Y., Milesi, P., Zhou, L., Chen, J., Li, L., Cui, B., Niu, S., Westin, J., Karlsson, B., García-Gil, M. R., Lascoux, M., & Wu, H. X. (2021). Leveraging breeding programs and genomic data in Norway spruce (Picea abies L. Karst) for GWAS analysis. *Genome Biology*, *22*(1), 179. https://doi.org/10.1186/s13059-021-02392-1

Cingolani, P., Platts, A., Wang, L. L., Coon, M., Nguyen, T., Wang, L., Land, S. J., Lu, X., & Ruden, D. M. (2012). A program for annotating and predicting the effects of single nucleotide polymorphisms, SnpEff: SNPs in the genome of Drosophila melanogaster strain w1118; iso-2; iso-3. *Fly*, *6*(2), 80–92. https://doi.org/10.4161/fly.19695

Coop, G., Witonsky, D., Di Rienzo, A., & Pritchard, J. K. (2010). Using Environmental Correlations to Identify Loci Underlying Local Adaptation. *Genetics*, *185*(4), 1411–1423. https://doi.org/10.1534/genetics.110.114819

Durand, E., Jay, F., Gaggiotti, O. E., & François, O. (2009). Spatial Inference of Admixture Proportions and Secondary Contact Zones. *Molecular Biology and Evolution*, *26*(9), 1963–1973. https://doi.org/10.1093/molbev/msp106

Günther, T., & Coop, G. (2013). Robust Identification of Local Adaptation from Allele Frequencies. *Genetics*, *195*(1), 205–220. https://doi.org/10.1534/genetics.113.152462

Jaramillo-Correa, J. P., Verdú, M., & González-Martínez, S. C. (2010). The contribution of recombination to heterozygosity differs among plant evolutionary lineages and life-forms. *BMC Evolutionary Biology*, *10*(1), 22. https://doi.org/10.1186/1471-2148-10-22

Karunarathne, P., Zhou, Q., Schliep, K., & Milesi, P. (2023). A comprehensive framework for detecting copy number variants from single nucleotide polymorphism data: 'rCNV', a versatile r package for paralogue and CNV detection. *Molecular Ecology Resources*, *23*(8), 1772–1789. https://doi.org/10.1111/1755-0998.13843

Kass, R. E., & Raftery, A. E. (1995). Bayes Factor. *Journal of the American Statistical Association*, *90*(430), 773–795.

Lê, S., Josse, J., & Husson, F. (2008). FactoMineR: An R Package for Multivariate Analysis. *Journal of Statistical Software*, *25*, 1–18. https://doi.org/10.18637/jss.v025.i01

Leal, J. L., Milesi, P., Hodková, E., Zhou, Q., James, J., Eklund, D. M., Pyhäjärvi, T., Salojärvi, J., & Lascoux, M. (2024). Complex Polyploids: Origins, Genomic Composition, and Role of Introgressed Alleles. *Systematic Biology*, *73*(2), 392–418. https://doi.org/10.1093/sysbio/syae012

Li, H. (2013, March 16). *Aligning sequence reads, clone sequences and assembly contigs with BWA-MEM*. arXiv.Org. https://arxiv.org/abs/1303.3997v2

Li, H., & Durbin, R. (2009). Fast and accurate short read alignment with Burrows–Wheeler

transform. *Bioinformatics*, *25*(14), 1754–1760. https://doi.org/10.1093/bioinformatics/btp324

Li, H., Handsaker, B., Wysoker, A., Fennell, T., Ruan, J., Homer, N., Marth, G., Abecasis, G., Durbin, R., & 1000 Genome Project Data Processing Subgroup. (2009). The Sequence Alignment/Map format and SAMtools. *Bioinformatics*, *25*(16), 2078–2079. https://doi.org/10.1093/bioinformatics/btp352

Li, L., Milesi, P., Tiret, M., Chen, J., Sendrowski, J., Baison, J., Chen, Z., Zhou, L., Karlsson, B., Berlin, M., Westin, J., Garcia-Gil, M. R., Wu, H. X., & Lascoux, M. (2022). Teasing apart the joint effect of demography and natural selection in the birth of a contact zone. *New Phytologist*, *236*(5), 1976–1987. https://doi.org/10.1111/nph.18480

Luu, K., Bazin, E., & Blum, M. G. B. (2017). pcadapt: An R package to perform genome scans for selection based on principal component analysis. *Molecular Ecology Resources*, *17*(1), 67–77. https://doi.org/10.1111/1755-0998.12592

Marcus, J., Ha, W., Barber, R. F., & Novembre, J. (2021). Fast and flexible estimation of effective migration surfaces. *eLife*, *10*, e61927. https://doi.org/10.7554/eLife.61927

McKenna, A., Hanna, M., Banks, E., Sivachenko, A., Cibulskis, K., Kernytsky, A., Garimella, K., Altshuler, D., Gabriel, S., Daly, M., & DePristo, M. A. (2010). The Genome Analysis Toolkit: A MapReduce framework for analyzing next-generation DNA sequencing data. *Genome Research*, *20*(9), 1297–1303. https://doi.org/10.1101/gr.107524.110

Milesi, P., Kastally, C., Dauphin, B., Cervantes, S., Bagnoli, F., Budde, K. B., Cavers, S., Fady, B., Faivre-Rampant, P., González-Martínez, S. C., Grivet, D., Gugerli, F., Jorge, V., Lesur Kupin, I., Ojeda, D. I., Olsson, S., Opgenoorth, L., Pinosio, S., Plomion, C., … Pyhäjärvi, T. (2024). Resilience of genetic diversity in forest trees over the Quaternary. *Nature Communications*, *15*(1), 8538. https://doi.org/10.1038/s41467-024-52612-y

Niu, S., Li, J., Bo, W., Yang, W., Zuccolo, A., Giacomello, S., Chen, X., Han, F., Yang, J., Song, Y., Nie, Y., Zhou, B., Wang, P., Zuo, Q., Zhang, H., Ma, J., Wang, J., Wang, L., Zhu, Q.,

… Wu, H. X. (2022). The Chinese pine genome and methylome unveil key features of conifer evolution. *Cell*, *185*(1), 204-217.e14. https://doi.org/10.1016/j.cell.2021.12.006

Nystedt, B., Street, N. R., Wetterbom, A., Zuccolo, A., Lin, Y.-C., Scofield, D. G., Vezzi, F., Delhomme, N., Giacomello, S., Alexeyenko, A., Vicedomini, R., Sahlin, K., Sherwood, E., Elfstrand, M., Gramzow, L., Holmberg, K., Hällman, J., Keech, O., Klasson, L., … Jansson, S. (2013). The Norway spruce genome sequence and conifer genome evolution. *Nature*, *497*(7451), 579–584. https://doi.org/10.1038/nature12211

Pyhäjärvi, T., Kujala, S. T., & Savolainen, O. (2019). 275 years of forestry meets genomics in Pinus sylvestris. *Evolutionary Applications*, *13*(1), 11. https://doi.org/10.1111/eva.12809

R Core Team. (2025). *R: A Language and Environment for Statistical Computing*. R Foundation for Statistical Computing. https://www.R-project.org/

Salojärvi, J., Smolander, O.-P., Nieminen, K., Rajaraman, S., Safronov, O., Safdari, P., Lamminmäki, A., Immanen, J., Lan, T., Tanskanen, J., Rastas, P., Amiryousefi, A., Jayaprakash, B., Kammonen, J. I., Hagqvist, R., Eswaran, G., Ahonen, V. H., Serra, J. A., Asiegbu, F. O., … Kangasjärvi, J. (2017). Genome sequencing and population genomic analyses provide insights into the adaptive landscape of silver birch. *Nature Genetics*, *49*(6), 904–912. https://doi.org/10.1038/ng.3862

Sayols, S. (2023). rrvgo: A Bioconductor package for interpreting lists of Gene Ontology terms. *microPublication Biology*, *2023*, 10.17912/micropub.biology.000811. https://doi.org/10.17912/micropub.biology.000811

Vidalis, A., Scofield, D. G., Neves, L. G., Bernhardsson, C., García-Gil, M. R., & Ingvarsson, P. K. (2018). *Design and evaluation of a large sequence-capture probe set and associated SNPs for diploid and haploid samples of Norway spruce (Picea abies)* (p. 291716). bioRxiv. https://doi.org/10.1101/291716

Wang, K., Li, M., & Hakonarson, H. (2010). ANNOVAR: Functional annotation of genetic variants from high-throughput sequencing data. *Nucleic Acids Research*, *38*(16), e164.

https://doi.org/10.1093/nar/gkq603
